# The Vegetarian

**DOI:** 10.1192/pb.bp.116.055285

**Published:** 2017-04

**Authors:** Ginevra Read

This short novel, winner of the Man Booker International Prize 2016, is translated from Korean and tells the story of Yeong-hye, a young woman in modern day South Korea. It is a fascinating and thought-provoking narrative that starts with Yeong-hye choosing to become vegetarian. This seemingly unremarkable and straightforward choice turns out to be nothing of the sort – vegetarianism is almost unheard of in Korea but, more importantly, Yeong-hye is on the verge of serious mental illness. Yeong-hye's stance is challenged by all of those around her, but she remains steadfast. It transpires that becoming vegetarian is the prodromal seed of an all-encompassing psychosis which will take Yeong-hye on a journey from being vegetarian to believing that she is vegetal in nature and therefore food is superfluous to her needs.

Clinicians will be acquainted with the somewhat perplexing process by which this intricate and emotive story develops. Information appears not in neat chronological order but in a tangle that needs some work to unpick. Yeong-hye's story is told in three parts. We hear first from her husband, then her brother-in-law and finally her sister, all the while following the unravelling of Yeong-hye's internal and external world, in a tale that deepens in complexity and darkness as it unfolds. Interspersed italicised monologues allow us a brief glimpse of Yeong-hye's muddled (and muddling) mind. By portraying thoughts that mingle with dreams and memories in a way that confuses the reader as to what is real and what is not, Kang elegantly conveys something of Yeong-hye's mental state. We don't hear much about Yeong-hye's premorbid adult life, other than through her husband, who says she was ‘ordinary’ and functioned to his liking. We can, however, sense the weight of the oppression she is subject to and guess that although becoming vegetarian may have marked an important transition point in her illness, it is unlikely to have been the beginning of it. The husband's account of Yeong-hye's condition reveals, through the lens of his own narcissism, a shocking lack of concern for his wife beyond her role in satisfying his immediate needs. He views Yeong-hye as an object and a possession, and this is most apparent in his remorseless and matter-of-fact description of raping her. A meal with her husband's boss tells us something about society's inflexible expectations and demonstrates that the lack of compassion experienced by Yeong-hye is multifaceted. We see Yeong-hye's father in action and learn a little about her upbringing; as a result, the degree to which she has been repressed and forced to endure throughout her life becomes clearer, and the powerful, subversive resistance enacted through her illness begins to make sense.

The second part of the book is equally disturbing and leads us to the brother-in-law, a less than successful video-artist who becomes obsessed with Yeong-hye's pre-pubertal appearance and whose paraphilic behaviour uncomfortably exposes her vulnerability.

In the final part of the book, several years later, we join Yeong-hye's sister In-hye as she visits her in a psychiatric hospital. In-hye now faces the repercussions of preceding events and the resulting family disintegration. We hear more about the sisters' childhood and the abuse which they experienced; we learn that In-hye continues to suffer her own anguish as a corollary and that she in some way envies her sister's position.

This is an astonishing book. Strange, surreal and beautifully written. The idea that people could find themselves surrounded by such brutal inhumanity and lack of connection that they reject their current existence and instead opt for transformation into a life form that does not involve thought or feeling is indescribably sad, but probably not beyond imagination for most psychiatrists. Readers will find that they must piece together the jigsaw of Yeong-hye's life, and as hard as they try, the image is not clear and the final pieces can never be found – an experience to which most of us surely relate.

